# Trabecular Bone Score and Osteoprotegerin as Useful Tools in the Assessment of Bone Deterioration in Acromegaly

**DOI:** 10.3389/fendo.2022.862845

**Published:** 2022-04-21

**Authors:** Aleksandra Jawiarczyk-Przybyłowska, Jowita Halupczok-Żyła, Joanna Syrycka, Agnieszka Zembska, Justyna Kuliczkowska-Płaksej, Marek Bolanowski

**Affiliations:** Department and Clinic of Endocrinology, Diabetes and Isotope Therapy, Medical University, Wrocław, Poland

**Keywords:** TBS, BMD, osteoporosis, fracture, osteoprotegerin, acromegaly

## Abstract

**Purpose:**

This study aimed to assess bone mineral density (BMD) and trabecular bone score (TBS) in 61 patients from the acromegaly group (AG) with regard to the activity of the disease in comparison to 42 patients—control group (CG). We also analyzed selected bone markers and their association with BMD and TBS.

**Materials and Methods:**

Lumbar spine and femoral neck BMD measurements were performed. TBS values were obtained. Serum concentrations of selected bone markers, including osteoprotegerin (OPG), were measured.

**Results:**

We revealed a difference in TBS values between the AG and CG as well as between the TCA (treatment-controlled acromegaly) vs. CG and TCA+CA (cured acromegaly) vs. CG. We did not observe any statistically significant difference in BMD. OPG had a lower concentration in the CG compared to the AG. TBS correlated negatively with OPG in the AG (r = −0.31, p = 0.01) and in the TCA+ CA group (r = −0.3, p = 0.01).

**Conclusions:**

The acromegalic patients have altered bone microstructure as indicated by the decreased TBS regardless of the activity of the disease and BMD. OPG could be a marker of the destruction of the bone microstructure, but further studies are needed.

## Introduction

Acromegaly is an uncommon chronic disease, caused by excessive production of the growth hormone (GH) and the insulin-like growth factor 1 (IGF-1). It is characterized by typical and substantial facial and acral deformities as well as systemic disturbances, such as arthralgia, diabetes, hypertension, or cardiological disorders. The patients develop various complications leading to premature mortality (1, 2). One of them is osteoporosis and its consequences, which also have a negative influence on the quality of life of acromegalic patients (3). Patients with acromegaly have an increased risk of fractures, even when the bone mineral density (BMD) is within the normal range or slightly decreased (4, 5). It might be the result of insufficient quality of bone because of specific changes in bone microarchitecture (4–6).

It is known that in acromegaly the increased secretion of the GH and the IGF-1 leads to increased bone turnover (7, 8). Previous studies revealed that osteocalcin (OC), a specific marker of bone formation, is elevated in patients with active disease (8–10). Similarly, markers of bone resorption such as urinary hydroxyproline/creatinine, urinary type I collagen cross-linked N-telopeptide, and serum C-terminal collagen type I cross-links are higher as compared to healthy subjects (6, 8, 9, 11). Additionally, serum calcium and phosphate values and 24-h urinary calcium are increased in patients with active disease. Elevated serum calcium levels may be the consequence of increased intestinal calcium absorption. The high serum phosphate levels might be the result of increased intestinal and renal absorption, mediated by the GH (11, 12).

A new valuable tool in the evaluation of bone structure is trabecular bone score (TBS) measurement, which gives some information on bone microarchitecture based on a routine dual-energy X-ray absorptiometry (DXA) image of the lumbar spine (LS). This technique performs gray-level measurements on the DXA image of the LS, and in this way, it obtains information about trabecular microarchitecture (13). According to the previous studies, TBS provides information on the microarchitecture of bones and their quality. Studies showed that low TBS revealed widely spread and poorly connected trabeculae. On the other hand, high TBS is connected with a stronger trabecular structure (14). The high TBS result reflects better bone structure, whereas the low TBS indicates impaired bone structure associated with higher fracture risk (15). For now, we do not have established cutoff points for the obtained TBS to classify as normal or abnormal results. The available TBS range has only been established for postmenopausal women (14, 16, 17). Only a few studies assessed the TBS values in acromegaly patients. Low TBS values have been observed in patients with acromegaly and vertebral fractures (18). Hong et al. also showed low TBS values in acromegaly patients and suggest that it is a consequence of the elevated level of the GH as well as hypogonadism. The TBS values were decreased as compared to the control group (CG), while the BMD values were similar in both groups (19). Godang et al. revealed the decrease of bone markers [C-terminal telopeptides of type I collagen (CTX-1) and propeptide of type I procollagen (P1NP)] after treatment of acromegaly. In addition, they observed the increase of BMD, but TBS values did not change, so the risk of fracture persists despite the changes in BMD (13).

The main aim of this study was to assess the bone status (BMD and TBS values) in patients with acromegaly with regard to the activity of the disease and comparison to the CG. In addition, we analyzed the concentrations of human cross-linked N-telopeptide of type I collagen (NTX), OC, and osteoprotegerin (OPG) and their association with BMD and TBS. We also aimed to evaluate if there is an association between the activity of acromegaly and bone status.

## Materials and Methods

### Subjects

The study population consisted of 61 acromegaly patients (35 women and 26 men, mean age 60.92 ± 11.0 years) and 42 healthy subjects as the CG (25 women and 17 men, mean age 56.50 ± 11.2 years). All participants were recruited from the Department of Endocrinology, Diabetes and Isotope Therapy, Wroclaw Medical University. According to the Polish recommendation on diagnostic and therapeutic management of acromegaly, the study group was divided into 3 subgroups [active acromegaly (AA), treatment-controlled acromegaly (TCA), and cured acromegaly (CA)] (20). The AA group consisted of patients with IGF-1 higher than the sex- and age-matched normative reference values and nadir GH above 1 µg/L (ng/ml) during the oral glucose tolerance test (OGTT) (8 subjects). Among them, four of the patients had acromegaly *de novo*, before the treatment. The patients were assigned to the TCA group (35 subjects), during the treatment with long-acting somatostatin analogs, due to unsuccessful surgery. The patients after successful surgical treatment, with normal IGF-1 values and nadir GH below 1 µg/L (ng/ml) during the OGTT, were allocated to the CA group (18 subjects). The CG comprised 42 healthy individuals. Treatment characteristics of the study groups are listed in **Table 1**. Three types of analysis were performed in the classifications for the study. The first was done based on the activity of the disease (AA, CA, TCA, and CG). Second, the differences among the AA group, patients with both cured and controlled disease (TCA+CA), and the CG, were analyzed. The third step was to compare the patients with acromegaly (AA+TCA+CA) and the controls.

**Table 1:** Characteristics of acromegaly patients and the control group.

Group	AA	TCA	CA	CG	TCA+CA	AA+TCA+CA
Number	8	35	18	42	53	61
Age (years)	59.6 ± 11.6	61.8 ± 9.8	57.6 ± 11.6	56.5 ± 11.2	60.3 ± 10.5	60.2 ± 10.6
Body mass (kg)	91.8 ± 14.4	80.3 ± 15.8	81.1 ± 16.5	81.0 ± 13.4	81.3 ± 15.7	82.7 ± 15.8
Height (cm)	175.3 ± 13.1	166.4 ± 8.7	168.0 ± 7.1	169.0 ± 8.4	166.9 ± 8.2	168.0 ± 9.3
BMI (kg/m^2^)	29.9 ± 4.3	29.0 ± 4.6	27.8 ± 5.1	28.8 ± 4.7	28.95 ± 4.6	29.0 ± 4.6
IGF-1 (ng/ml)	332.75 ± 146.0*#^$	131.28 ± 35.4	144.56 ± 51.96*	113.8 ± 26.45	136.8 ± 41.8*	162.5 ± 91.9*
IGF-1/ULN	1.5 ± 0.79*#^$	0.62 ± 0.17*	0.63 ± 0.25*	0.51 ± 0.14	0.63 ± 0.18*	0.75 ± 0.44*
Vitamin D (ng/ml)	32.2 ± 21.0	24.31 ± 10.4	26.3 ± 12.3	24.5 ± 6.4	25.0 ± 11.0	26.0 ± 12.8
Calcium (mg/dl)	9.5 ± 0.3	9.3 ± 0.4	9.2 ± 0.4	9.3 ± 0.2	9.3 ± 0.4	9.3 ± 0.4
Phosphate (mg/dl)	3.6 ± 0.4	3.2 ± 0.5	3.3 ± 0.5	3.1 ± 0.4	3.2 ± 0.5	3.3 ± 0.5*
Osteoprotegerin (pmol/L)	4.5 ± 0.9	5.48 ± 1.6	3.9 ± 1.3	3.7 ± 1.1	4.9 ± 1.7	4.9 ± 1.65
Lumbar spine TBS	1.25 ± 0.10	1.19 ± 0.12*	1.27 ± 0.16	1.29 ± 0.10	1.21 ± 0.10*	1.22 ± 0.13*

AA, active acromegaly; TCA, treatment controlled acromegaly; CA, cured acromegaly; CG, control group; TBS, trabecular bone score; BMI, body mass index; IGF-1, insulin-like growth factor 1; ULN, upper limit of the normal range.

^*^vs. CG, ^#^vs. TCA, ^^^vs. CA, ^&^vs. AA, ^$^vs. TCA+CA, significant differences (p < 0.05).

Patients’ weight (kg) and height (m) were measured, and body mass index (BMI) was calculated. The medical histories were taken and included information such as clinical risk factors mentioned in the FRAX algorithm (exogenous glucocorticoids, alcohol intake, previous fracture, fracture of the hip in the parent, rheumatoid arthritis, secondary osteoporosis, and smoking habits), drug history, and reproductive status.

### Bone Mineral Density and Trabecular Bone Score Measurements

The BMD of the LS (L1–L4) and the femoral neck was measured using the DXA method by Horizon A Hologic densitometer and was expressed in absolute values (grams per square centimeter) as well as SD from the peak bone mass (T-score) and the expected mass for the age-matched population (Z-score). In the postmenopausal women and men ≥50 years of age, the diagnosis of osteoporosis was made on the BMD T-score ≤−2.5. Low bone mass was diagnosed as a T-score <−1.0 and >−2.5, and values of T-score ≥−1.0 were normal bone density. In the premenopausal women and men <50 years of age, the value of BMD was expressed as a Z-score. Z-score values of ≤−2.0 should be categorized as “below the expected range for age” and those with Z-score >−2.0 as “within the expected range for age” (21, 22).

All TBS values were analyzed using the TBS iNsight software (v 3.0.3.0, Medimaps, Pessac, France) using LS BMD DXA files from the database. TBS exams were performed by the same author. In the previous study, the following range for TBS values for postmenopausal women has been proposed: TBS ≥ 1.350 is considered normal, TBS between 1.200 and 1.350 is considered to be consistent with partially degraded microarchitecture, and TBS ≤ 1.200 is considered degraded microarchitecture (17). These cutoff points were accepted by authors from other countries who used TBS values in their publications. In this study, the criteria proposed in the meta-analysis prepared by McCloskey et al. were used to define fracture risk TBS thresholds <1.23 and >1.32 for the highest and lowest risk, respectively. The intermediate risk is defined by TBS of 1.23–1.31 (23–25).

### Laboratory Examinations

The GH and IGF-1 concentrations were assayed using a chemiluminescence immunometric assay (Immulite 2000, Siemens, Tarrytown, NY, USA). Vitamin D was assessed by chemiluminescence immunometric assay (Architect i1000, Abbott Laboratories, Abbott Park, IL, USA; limit of quantitation (LOQ), 2.4 ng/ml). Vitamin D deficiency was diagnosed at a concentration < 20 ng/ml, suboptimal status 20–30 ng/ml, and optimal status 30–70 ng/ml. Serum calcium, inorganic phosphate, magnesium, and alkaline phosphatase were measured using colorimetric assays on an Architect c4000 (Abbott Laboratories, USA).

Serum concentrations of OPG, OC, and NTX were determined by ELISA using commercially available kits purchased from various suppliers. Serum was separated by centrifugation of the blood samples at 2,000 × *g* for 15 min. Serum samples were aliquoted and stored at −70°C. Samples were brought to room temperature before starting the analysis. The following kits were used: OPG ELISA (Catalogue No. BI-20403) from Biomedica (Vienna, Austria), N-MID Osteocalcin ELISA (Catalogue No. AC-11PL-A) from Immunodiagnostic Systems (Boldon, UK), and human cross-linked N-telopeptide of type I collagen (Catalogue No. E1383Hu) from Bioassay Technology Laboratory (Shanghai, China). Assays were carried out according to the manufacturers’ instructions. The samples were assayed in duplicate.

The local bioethics committee approved the protocol of the study. All subjects gave written informed consent in accordance with the Declaration of Helsinki.

### Statistical Analysis

The statistical analysis was performed using R for Windows statistical software (version 4.0.4, Vienna, Austria). Variables were presented as mean ± SD. The Shapiro–Wilk test was used to check the normality of the data. The Mann–Whitney test (for two groups) or Kruskal–Wallis test (for more than two groups) was applied to compare quantitative variables. Categorical variables were compared by the chi-square test or Fisher’s exact test. Correlations between parameters were calculated using Spearman’s rank correlation test. Multiple regression analysis was used to indicate the predictors of TBS value. p-Values <0.05 were considered significant.

## Results

The general characteristics of the study group and the CG are presented in **Table 2**. According to the DXA results, osteoporosis was diagnosed in 3 patients from the CG and 7 from the acromegaly group (AG) and low bone mass in 14 patients from the CG and 23 from the AG; normal bone mass was recorded in 24 in the CG and 31 patients in the AG. The highest risk of fractures (TBS < 1.23) was observed more frequently in the AG (34 patients; 55.5%) compared to the CG (12 patients, 28.5%) (p = 0.012). The lowest risk of fractures (TBS > 1.32) was in 15 (25%) of acromegaly patients and 17 (40.5%) of controls. The intermediate risk defined by TBS 1.23–1.31 was in 12 (19.5%) patients from the AG and 13 (41%) from the CG. Fragility fractures were identified in four acromegaly patients, including vertebral, femoral neck, arm, and hand fractures (**Table 3**). There were no low-trauma fractures in the CG. Traumatic fractures were recorded in nine patients with acromegaly and ten controls.

**Table 2:** Characteristics of the study group and control group—treatment.

Number of patients using the drug	Octreotide LAR (intramuscularly, every 28 days)				Lanreotide LAR (subcutaneously, every 28 days)	Pasireotide LAR (intramuscularly, every 28 days)
	10 mg	20 mg	30 mg	40 mg	120 mg	40 mg
AA			1	1	2	
TCA	3	2	6	4	16	4
CG						
Other drugs						
	Hydrocortisone	l-Thyroxine	Testosterone	Vitamin D3	Bisphosphonate	Calcium
Study group (AA, TCA, CA)	10	24	1	19	3	8
CG		5		2		1

AA, active acromegaly; TCA, treatment controlled acromegaly; CA, cured acromegaly; CG, control group.

**Table 3:** Characteristics of patients with non-traumatic fractures.

Patient	Age (years) gender, and group	Clinical risk factors	Lumbar spine TBS	Lumbar spine BMD (g/cm^2^)	T-score	Femoral neck BMD (g/cm^2^)	T-score	Osteoprotegerin (pmol/L)
1	73 years Female TCA	Hip fractures in mother	0.984	0.725	−2.9	0.604	−2.2	9.321
2	72 years Male TCA	ACTH deficiency hypogonadism	1.25	1.33	−0.4	0.814	−0.9	7.841
3	69 years Female TCA		1.134	0.916	0.9	0.821	0.2	8.204
4	75 years Female AA		1.186	0.88	0.9	0.604	−2.2	5.88

AA, active acromegaly; TCA, treatment controlled acromegaly; TBS, trabecular bone score; BMD, bone mineral density; ACTH, adrenocorticotropic hormone.

**Figure 1:**
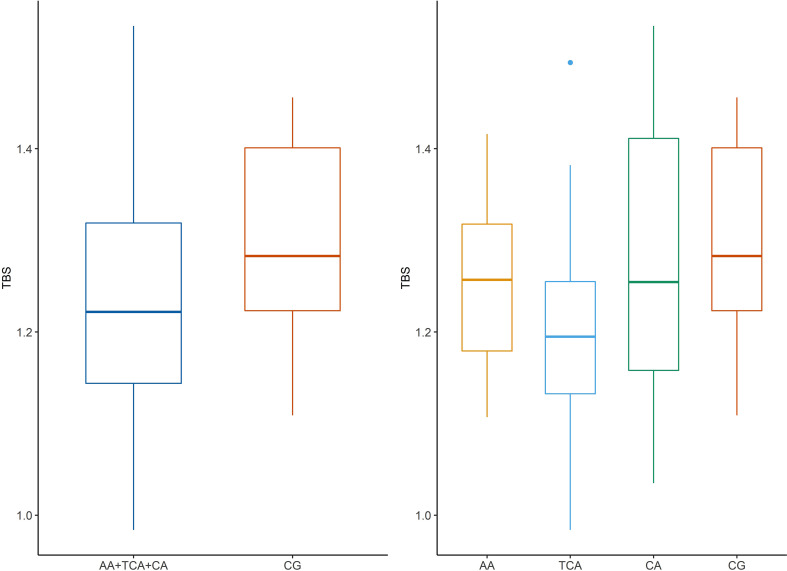
Trabecular bone score (TBS) in patients in acromegaly group compared to control group. AA, active acromegaly; TCA, treatment controlled acromegaly; CA, cured acromegaly; CG, control group; TBS, trabecular bone score.

Among AGs, the CA had the highest value of TBS, and the TCA group had the smallest. We observe a statistically significant difference of TBS values between the whole AG and CG (p = 0.0019) as well as between the TCA group vs. CG (p = 0.023) and the both cured and controlled disease vs. the CG (p = 0.0072). The highest value of TBS was in the CG (**Figure 1**). We did not observe any statistically significant difference in lumbar total BMD as well as femoral neck BMD among the groups, regardless of used division.

There were no statistically significant differences in results of DXA and TBS between women and men, regardless of the group division.

In the whole AG, the analysis revealed a significant negative correlation between TBS and age as well as BMI (r = −0.39, p = 0.001). Moreover, negative correlations were also observed in the both cured and controlled disease (r = −0.36, p = 0.007) in the CA group (r = −0.55, p = 0.01) and the CG (r = −0.56, p = 0.000). In the whole AG as well as in the both cured and controlled disease group and CA group, we noticed a positive statistically significant correlation of TBS with IGF-1 (r = 0.39, p = 0.001; r = 0.40, p = 0.002; and r = 0.61, p = 0.006, respectively). In the AA group, we observed a tendency to a negative correlation between these two parameters, but it was not statistically significant. The statistically significant positive correlations of TBS with the values of BMD, T-score, and Z-score were observed (**Table 4**).

**Table 4:** The correlations of TBS with BMD, T-score, and Z-score in all study groups.

Group	AA	TCA	CA	CG	AA+TCA+CA	TCA+ CA
Lumbar spine T-score	R = 0.85 **p = 0.010**	R = 0.49 **p = 0.002**	R = 0.79 **p = 0.000**	R = 0.51 **p = 0.000**	R = 0.63 **p = 0.000**	R = 0.60 **p = 0.000**
Lumbar spine Z-score	R = 0.89 **p = 0.002**	R = 0.35 **p = 0.035**	R = 0.66 **p = 0.002**	R = 0.05 p = 0.71	R = 0.48 **p = 0.000**	R = 0.43 **p = 0.001**
Lumbar spine BMD	R = 0.49 **p = 0.000**	R = 0.49 **p = 0.000**	R = 0.76 **p = 0.000**	R = 0.49 **p = 0.000**	R = 0.60 **p = 0.000**	R = 0.57 **p = 0.000**
Femoral neck T-score	R = 0.88 **p = 0.007**	R = 0.3 p = 0.079	R = 0.68 **p = 0.001**	R = 0.36 **p = 0.019**	R = 0.496 **p = 0.000**	R = 0.44 **p = 0.000**
Femoral neck Z-score	R = 0.97 **p = 0.000**	R = 0.13 p = 0.433	R = 0.47 **p = 0.046**	R = 0.12 p = 0.429	R = 0.35 **p = 0.004**	R = 0.26 p = 0.05
Femoral neck BMD	R = 0.83 **p = 0.015**	R = 0.83 **p = 0.015**	R = 0.67 **p = 0.002**	R = 0.36 p = 0.018	R = 0.43 **p = 0.000**	R = 0.36 **p = 0.006**

Bold values = significant differences (p < 0.05).

AA, active acromegaly; TCA, treatment controlled acromegaly; CA, cured acromegaly; CG, control group; BMD, bone mineral density; TBS, trabecular bone score.

We did not find any statistically significant difference in NTX and OC concentrations, regardless of used division (data not shown). OPG had a statistically significantly lower concentration in the CG compared to the whole AG as well as the TCA+CA and TCA. The concentration of OPG was also significantly higher in the treatment-controlled group compared to the CA group (**Table 1**).

**Figure 2:**
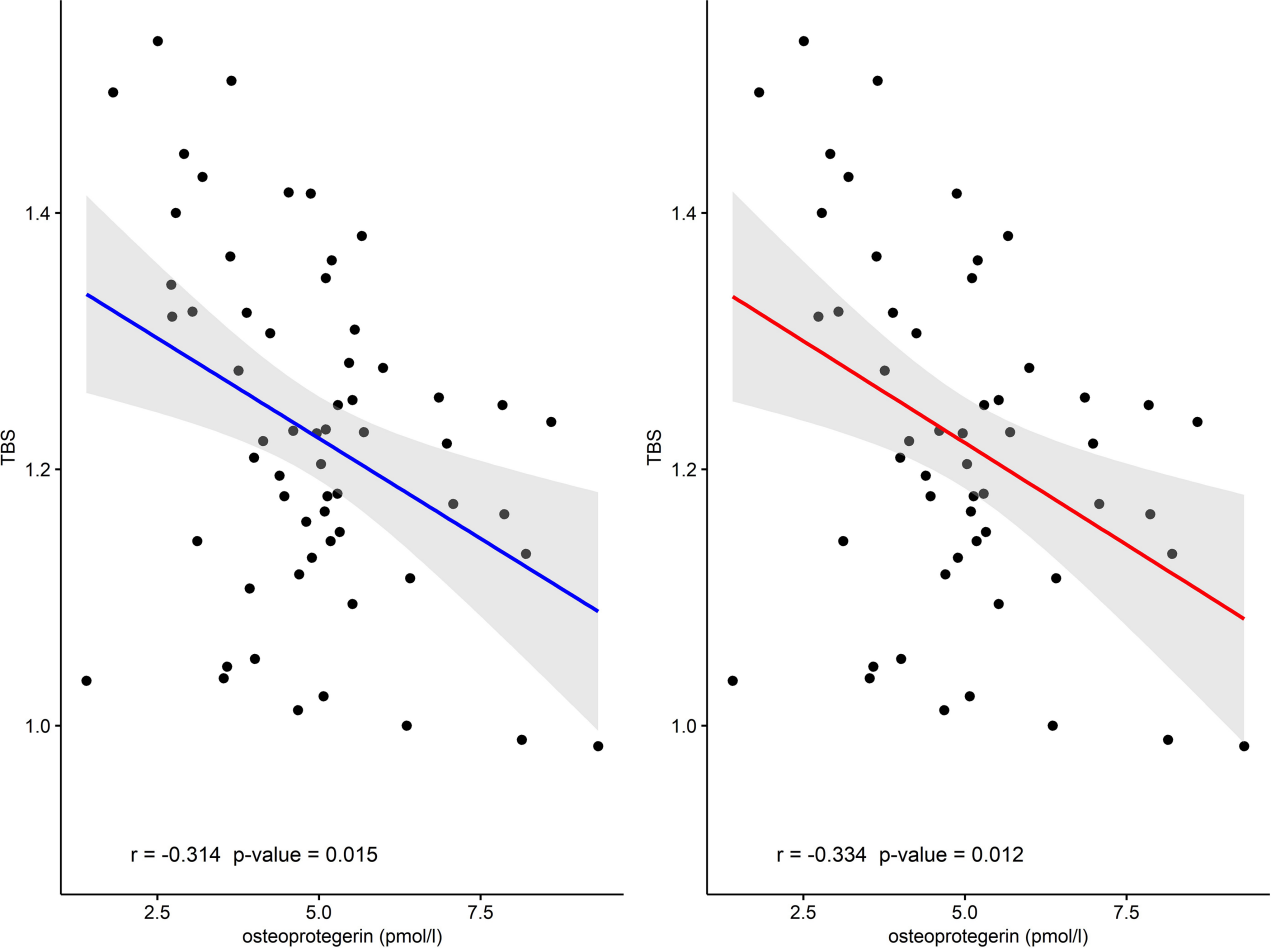
The correlation between TBS and osteoprotegerin in AA+TCA+CA group (r = −0.314) and in the TCA+CA group (r = −0.334). AA, active acromegaly; TCA, treatment controlled acromegaly; CA, cured acromegaly; TBS, trabecular bone score.

In this study, we did not find any correlation between TBS and NTX as well as OC concentrations (data not shown), although we revealed some association between TBS and OPG levels. Statistically significant negative correlations were observed between these two parameters in the whole AG (r = −0.314, p = 0.015) as well as in both the cured and controlled disease group (r = −0.334, p = 0.012) (**Figure 2**). This negative correlation was also statistically significant in the CG (r = −0.407, p = 0.007).

A multivariate linear regression gathering the most important independent factors was performed to find essential parameters that influence the TBS values among patients harboring acromegaly. The first model included the following factors: age, sex, height, body mass, IGF-1, estradiol, and prolactin. The strongest factors that determined TBS value were age (β = −0.004, p = 0.0078) and IGF-1 (β = 0.0004, p = 0.0484). The second model comprised sex, height, body mass, OPG, IGF-1, estradiol, and prolactin. The significant factor was OPG (β = −0.025, p = 0.015).

We did not observe any differences of 25(OH)D3 levels among the tested groups. A significant correlation between 25(OH)D3 levels and TBS value was not found. Similarly, we did not find any correlation between TBS and calcium, inorganic phosphorus, and magnesium levels (data not shown).

## Discussion

The obtained results confirmed that patients with acromegaly had lower TBS values. It is known that DXA measurements reflect the total area density (trabecular and cortical) and bone quantity. The risk of fractures could be underestimated by BMD because in acromegaly the bone quality is also disturbed. Using the Fracture Risk Assessment Tool (FRAX) in acromegaly is not validated; however, we do not have enough useful tools to assess the real fracture risk. So it is very important to find new markers and tools for estimating the risk for osteoporotic fractures. In our studies, we did not observe any statistically significant difference of LS BMD as well as femoral neck BMD among the groups regardless of the division used. According to the TBS values, the majority of acromegaly patients had degraded microarchitecture and consequently were at high risk of sustaining a fracture. The previous study showed that the GH and the IGF-1 have different influences on trabecular and cortical bone, and they are differently distributed in these localizations (26). Besides, the bone quality depends not only on bone density and bone turnover but also on the macro- and microstructures of bone as well as the collagen integrity (27). Trabecular bone is more prone to GH excess, which leads to impairment of the structural integrity of the trabecular bone. The disruption of trabecular bone is caused by the decreased trabecular number and increased trabecular separation. For these reasons, fractures could be present in acromegaly patients with normal or slightly decreased BMD (11, 28, 29). Hong et al. demonstrated a significantly decreased TBS in AA compared to healthy subjects, although BMD at all sites was similar for the two groups (19). Similarly, Sala et al. showed that patients with AA had a significantly lower TBS compared to control, regardless of age, sex, BMI, and gonadal status (30). Correspondingly, Calatayud et al. revealed significantly lower TBS values in controlled acromegalic patients, despite no difference in LS BMD (31). In another study, Goldang et al. showed that treatment of acromegaly influences TBS and BMD differently. They observed the decrease of TBS and the increase of BMD following the treatment (13). On the contrary, in the recent study, no TBS and BMD variation was observed 1 year after the biochemical cure or control of the acromegaly disease (30).

In earlier studies, the analysis of TBS concerned active or controlled acromegaly, whereas our study comprises and compares TBS values in patients with active, controlled, and cured acromegaly. In the TCA group as well as in the TCA+ CA group, TBS was decreased in comparison to controls, while BMD was similar among these groups. These results confirmed that TBS and BMD reflect different features of bone and explain the incident of fractures in acromegaly, despite the normal BMD values (28, 29). This can be explained by the fact that lower TBS value reflects impaired bone microstructure, which is associated with higher fracture risk. The smallest TBS values in the TCA group may indicate that patients with controlled acromegaly are still prone to fractures. There is a need for long-term monitoring of the bone condition.

GH and IGF-1 have a significant role in the regulation of bone metabolism. During the pubertal period, GH stimulates longitudinal bone growth, whereas adolescence and early adulthood regulate skeletal maturation until the achievement of bone peak mass. Additionally, in adulthood, GH regulates bone turnover and, in this way, maintains bone mass. Similarly, IGF-1 plays an essential role in the growth of the long bone, the maturation of the skeleton, and the maintenance of bone mass not only during growth but also during late adulthood and aging (32). Several previous studies showed that GH deficiency is responsible for the decrease in bone mass and osteoporosis (12, 33), but the influence of GH excess on bone status is not clear. In acromegaly, the status of bone mass and quality could be the consequence of some other factors, for example, hypogonadism, hyperprolactinemia, or disease activity (12). The anabolic effects of GH and IGF-1 are the results of the stimulation of the proliferation and differentiation of osteoblasts and the formation of the extra-cellular matrix. On the other hand, they stimulate the resorptive activity of osteoclasts (12). In our studies, we observed that TBS correlated significantly positively with IGF-1 in the whole AG as well as in the TCA+CA and CA groups. The previous study has shown that LS BMD is positively correlated with IGF-1 levels (34). These results confirmed the anabolic impact of IGF-1 on bone (35). Another study showed that IGF-1 burden (IGF-1 level x disease duration) correlated negatively with BMD Z-score at the LS, and the authors suggest that IGF-1 burden could be an excellent indicator of BMD (36). The long-term exposure to overproduction of GH and IGF-1 could play a pathophysiological role in the development of IGF-I receptor resistance. The development of IGF-1 receptor resistance could contribute to the reduction of the activity of osteoblasts during acromegaly remission (37).

In the present study, we observed an inverse association between TBS value and age. This finding is in accordance with results from previous large cross-sectional studies involving cohorts of French, Canadian, African American, Lebanese, Thai, and Japanese subjects (38). Dufour et al. reported a linear decrease of approximately 14.5% in L1–L4 TBS values over 40 years (between 45 and 85 years of age) in a cohort of 5,942 French women. Moreover, after 65 years old, an acceleration of the TBS value decrease was presented. Similar to our results, TBS values correlated negatively with BMI and did not correlate with height (23).

In our study, we also analyzed selected bone markers and their associations with TBS. We did not find any statistically significant difference in NTX and OC concentrations, regardless of the used division. We did not observe the correlation between TBS as well as BMD and bone markers (NTX and OC concentrations). In some previous studies, an increase of bone turnover markers (OC and CTX-1) in AGs compared to controls (10, 30) or CA (39) was demonstrated, but they also did not find the correlation between these markers and BMD (10). Moreover, taking into account the fact that only 15% of OC is detected in the circulation, and that the characteristic feature of this non-collagenic protein is the high concentration variation in the circadian secretion profile (up to 30%) (40), this could have influenced the results obtained in our study and, as a result, the lack of significant differences in the concentration of OC between the groups. In addition, this molecule is unstable, and its half-life is only from several minutes to 1 h (40), which may also influence the determination of the concentration of this protein. For the same reason, we may not have observed the correction between TBS and OC concentration in the AG compared to the CG. In addition, it would seem that in our study, we will prove a positive correlation between the concentration of NTX and TBS. Perhaps the lack of this dependence was due to the determination of this protein in serum, and not in 24-h urine collection. This protein, like the aforementioned OC, is also characterized by daily variability, and therefore perhaps a better biological material would be 24-h urine collection (39), and our results could be comparable to those obtained by other researchers (41, 42). Some authors demonstrated that GH replacement therapy could induce a significant increase in OPG in plasma as well as in cortical and trabecular bone (43, 44). For these reasons, the influence of GH on human osteoblast-like cells in primer culture was studied, and it was shown that GH stimulates the secretion and expression of OPG in these cells. Moreover, the study showed that a specific antagonist of GH receptor (Pegvisomant) could prevent the increase of OPG, which confirmed that the influence of GH is direct receptor-mediated, and independent of IGF-1 (45). In the current research, OPG concentration was significantly higher in the whole AG as well as the CTA+CA and CTA compared to the CG. Similarly, Valassi et al. observed a greater concentration of OPG in controlled acromegaly patients (46). However, there are also studies that showed no statistically significant differences in OPG concentrations between patients with acromegaly and nonfunctioning pituitary adenomas or healthy individuals (47, 48). These discrepancies indicate the need for further research concerning the role of the OPG/RANKL system in bone remodeling in acromegaly. OPG, a secreted glycoprotein, has been identified as an osteoblast-derived regulator of bone resorption and is involved in maintaining bone mass (44). OPG acts by neutralizing the receptor activator of nuclear factor-kB ligand (RANKL), which is a crucial factor required for osteoclast differentiation and activation (45). OPG binding to RANKL inhibits osteoclast differentiation and bone resorption, which makes the RANKL/OPG ratio play a crucial role in bone turnover (48). Thereby, we hypothesize that an inverse association between OPG concentration and TBS values observed in our study may reflect a compensatory mechanism to degrade or partially degrade bone microarchitecture.

### Strengths and Limitations

Among the advantages and limitations of this study, a few things deserve to be emphasized. First of all, the studied population could be considered small, and this might explain especially the borderline differences among active AG and other groups. On the other hand, the size of the group seems to be adequate compared to the current literature, because acromegaly is a relatively rare disease. In this study, we confirmed and assessed the usefulness of TBS with regard to the activity of the disease.

As we know, various duration of the disease, different dosages, time spans, and the types of applied pharmacological treatment may affect the values of TBS and BMD. Due to the size of the groups, it was not possible to create another division, taking into account all these factors.

### Practical Implications of the Study/Future Interests

To sum up, this study confirmed that TBS could be a useful tool, which could predict the risk of fractures in acromegaly patients, independently of BMD. The acromegalic patients have altered bone microstructure as indicated by the decreased TBS and are at higher risk of fractures, regardless of the activity of the disease. TBS seems to be a very important analytical tool facilitating fracture risk assessment in those groups of patients. Creating an optimal tool for the evaluation of bone quality as soon as possible as prophylaxis of their irreversible consequences could result in the decreased risk of fractures in acromegaly. There is also a need to determine the association among some factors (TBS, serum levels of GH and IGF-1, duration of disease, age, gender, etc.) to conclude which are the most crucial and could be monitored to reduce the risk of fractures. OPG could be a marker of the destruction of bone microstructures, but more studies are needed. However, we did not demonstrate any correlation between the other two biochemical markers of bone turnover (NTX and OC concentrations) and TBS as well as BMD in the AG. We are sure that continuing research concerning this area is reasoned because it could be used to assess the dynamics of bone turnover in acromegaly. In addition, it could be a non-invasive tool to help monitor treatment compliance antiresorptive preparations, also used among patients with acromegaly.

### Conclusions

The acromegalic patients have altered bone microstructure as indicated by the decreased TBS regardless of the activity of the disease and BMD. OPG could be a marker of the destruction of the bone microstructure, but further studies are needed.

## Data Availability Statement

The raw data supporting the conclusions of this article will be made available by the authors, without undue reservation.

## Ethics Statement

The studies involving human participants were reviewed and approved by the Local Bioethics Committee Medical University, Wroclaw. The patients/participants provided their written informed consent to participate in this study.

## Author Contributions

AJ-P designed the project, provided the main conceptual ideas and proof outline, contributed to the interpretation of the results, compiled the literature sources, wrote the manuscript, and checked the references. JH-Z, JS, JK-P, and MB contributed to the conception and design of the study and helped in the collection, interpretation, and reference checking. AZ performed laboratory measurements and wrote a section of the manuscript. All authors contributed to the final version of the manuscript and approved it for publication.

## Funding

This study was supported by Statutory Activities by the Minister of Science and Higher Education (grant number ST. CT120.18.006 and SUB.C120.21.025).

## Conflict of Interest

The authors declare that the research was conducted in the absence of any commercial or financial relationships that could be construed as a potential conflict of interest.

## Publisher’s Note

All claims expressed in this article are solely those of the authors and do not necessarily represent those of their affiliated organizations, or those of the publisher, the editors and the reviewers. Any product that may be evaluated in this article, or claim that may be made by its manufacturer, is not guaranteed or endorsed by the publisher.
